# Elevated rates of dietary generalization in eusocial lineages of the secondarily herbivorous bees

**DOI:** 10.1186/s12862-023-02175-1

**Published:** 2023-11-20

**Authors:** T. J. Wood, A. Müller, C. Praz, D. Michez

**Affiliations:** 1 University of Mons, Research Institute for Biosciences, Laboratory of Zoology, Place du parc 20, 7000 Mons, Belgium; 2 ETH Zurich, Institute of Agricultural Sciences, Biocommunication and Entomology, Schmelzbergstrasse 9/LFO, 8092 Zurich, Switzerland; 3 University of Neuchâtel, Institute of Biology, Rue Emile-Argand 11, 2000 Neuchâtel, Switzerland; 4 InfoFauna – Swiss Zoological Records Center, Avenue de Bellevaux 51, 2000 Neuchâtel, Switzerland

**Keywords:** Host range, Specialization, Pareto distribution, Oligolecty, Phenology

## Abstract

**Background:**

Within the Hymenoptera, bees are notable for their relationship with flowering plants, being almost entirely dependent on plant pollen and nectar. Though functionally herbivorous, as a result of their role as pollinators, bees have received comparatively little attention as models for insect herbivory. Bees often display dietary specialization, but quantitative comparison against other herbivorous insects has not previously been conducted.

**Results:**

In the most comprehensive analysis to date for 860 bee species, dietary specialization amounted to 50.1% of studied species collecting pollen from between 1 and 2 botanical families with a relatively long tail of dietary generalists, with 11.1% of species collecting from more than 10 botanical families. This distribution deviated from the truncated Pareto distribution of dietary breadth seen in other herbivorous insect lineages. However, this deviation was predominantly due to eusocial bee lineages, which show a range of dietary breadths that conformed to a normal distribution, while solitary bees show a typical truncated distribution not strongly different from other herbivorous insects. We hypothesize that the relatively low level of dietary specialization in bees as a whole reflects the relaxation of the constraints typically observed in herbivorous insects with a comparatively reduced importance of plant chemistry and comparatively increased importance of phenology and foraging efficiency. The long flight periods of eusocial bees that are necessary to allow overlapping generations both allows and necessitates the use of multiple flowering resources, whereas solitary bees with short flight periods have more limited access to varied resources within a constrained activity period.

**Conclusions:**

Collectively, solitary bees show slightly lower specialization compared to other herbivorous insects, possibly due to their balanced relationship with plants, rather than direct antagonism such as seen in the direct consumption of plant tissues. An additional factor may be the mediocre diversity of bees at low latitudes combined with low levels of dietary specialization, whereas these areas typically display a high rate of specialization by herbivorous insects in general. Though the most important factors structuring dietary specialization in bees appear to differ from many other herbivorous insects, solitary bees show a surprisingly similar overall pattern of dietary specialization.

**Supplementary Information:**

The online version contains supplementary material available at 10.1186/s12862-023-02175-1.

## Background

Hymenoptera are one the major orders of insects, and thus represent one of the largest groups of animal life on Earth [1]. In addition to the huge number of described species, Hymenoptera display enormous diversity in life history including phytophagy, parasitism of arthropod and plant hosts, predatory behavior, nest building, eusociality, and the cultivation of fungi for food [2–5]. Within phytophagous Hymenoptera, a clear division can be made between the early branching sawfly lineages (Symphyta s.l.) that are ancestrally phytophagous, feeding on plant tissue such as leaves and wood [6] and the secondary phytophagy displayed by gall-forming parasitoid wasps (Cynipoidea), some ant lineages [7], one species of Crabronidae [8], and the pollen-feeding bees (Anthophila) and pollen wasps (Masarinae; [5]).

Use of pollen as a food source by Hymenoptera is most clearly and distinctively shown by the bees which emerged in the early to mid-Cretaceous period 110–140 million years ago, roughly concurrent with the emergence and diversification of flowering plants [9–11]. Almost all of the c. 20,000 described bee species feed their larvae exclusively on pollen and nectar, with three species reverting to carnivory [12] and a small number of lineages supplementing pollen provisions with floral oils [13]. Given the derived nature of the secondary herbivory in aculeate Hymenoptera, pollen feeding by bees has emerged after several major Hymenopteran adaptions, specifically the evolution of parasitoidism, the loss of the ovipositor/gain of a sting (Aculeata), the gain of nest making, and the gain of hunting and foraging behaviors [5]. As a result, pollinivory by the larval stage occurs within a constructed nest. Bee larvae are essentially immobile (though see [14]) and feed only on the pollen provisions collected by adult bees. As central place foragers, bees commute between nests and flower patches to gather pollen, nectar, and sometimes floral oils that collectively comprise the pollen provisions. This brood provisioning is found in all bee species, with the exception of brood parasitic bees. These species are parasites of other bee species, with their larvae usually killing the host larvae and consuming their provisions themselves [15].

Though bees are essentially herbivorous, since plant material represents the fundamental source of the protein available for their development (though see [16] for the role of microbial digestion, metabolism, and fermentation of pollen), they have received relatively little attention as models for studying herbivory in insects. This shortfall of study is apparent when compared to the rich literature available for other groups, particularly Lepidoptera (e.g. [17–22]). Bees have been more traditionally studied in the context of plant-pollinator interactions, concerning the transfer of pollen between individuals and populations, pollination syndromes, and specialization of floral visitation (e.g. [23–25]). The dichotomy between ‘pollinators’ and ‘herbivores’ persists in the literature (e.g. [26–28]). Though self-evidently there are reproductive benefits for plants resulting from bee-mediated pollination, the process of pollen harvesting and/or nectar robbing by bees can have negative impacts on plant fitness (e.g. [29]). Bee-plant interactions should consequently best be considered a balanced mutual exploitation [30, 31], contrasting the overwhelmingly negative effects of the direct consumption of plant tissues by insect herbivores [32–34]. The dietary niche of bees as herbivores has thus been somewhat obscured by their more visible ecological role as pollinators, as well as their nest building behavior which means that their herbivorous larval stage occurs hidden away from direct observation. Fundamentally however, bees remain herbivores.

Study of insect herbivory itself has produced a consensus that a large proportion of species are highly restricted and specialized in their use of host plants, typically utilizing only a single botanical family [17, 19, 22]. Ground-breaking quantitative analysis [22] has demonstrated that the distribution of specialization to generalization is highly unequal, with specialized species predominating (around 75%) with a long thin tail of increasingly generalized species. Whilst it might be hypothesized that insect herbivores display a range of diet breadths with a distribution conforming to normality, or indeed could display a bimodal distribution of diet breadths if intermediate specialization was maladaptive. However, the observed pattern of insect herbivore diets is that of a distribution dominated by specialists with a long tail of generalists [22]. This is best fit by a discrete truncated Pareto distribution (power-law probability distribution) rather than geometric or Poisson distributions [22]. The Pareto distribution is best described using the *α* parameter, as it is more informative than measures such as the mean given the highly non-symmetrical dietary distributions seen in insect herbivores, with a higher value indicating a higher dominance of specialists (*α* = 1 indicating a typical 80–20 Pareto distribution) and low values (for example, *α* < 0.30) increasingly indicating deviation from a Pareto distribution.

Whilst covering most groups of herbivorous insects globally [22], no data from bees were included in this analysis, making comparisons between these derived herbivores and the majority of insect herbivores difficult. Though many authors have recognized dietary specialization in bees going back to the nineteenth century (e.g. [35]), the study of both species-specific strategies and broader patterns has been plagued by the lack of quantitative data. However, an increasing number of quantitative studies focusing on pollen use from the perspective of individual bee species have been produced during the past 30 years, allowing assessments of relative dietary breadth within and between bee lineages e.g. [36–44].

This increased focus on pollen use by bees provides the opportunity to quantitatively assess dietary breadth in this secondarily herbivorous lineage, and to make explicit comparisons with trends in better-studied lineages (e.g. [22]) through measuring the extent to which the distribution of bee diet breadths conforms to the Pareto distribution. Given the vast period between the loss of herbivory by the most recent common ancestor of the sawfly family Cephidae and the clade Apocrita+Orussidae in the mid-Triassic [5], the reacquisition of phytophagy by bees (early Jurassic to the early to mid-Cretaceous, a period of at least 120 million years), and the commensurate changes in life history traits that occurred during this time, it could be expected that bees display patterns of phytophagy that differ meaningfully from lineages that have remained herbivorous throughout a much longer period of evolutionary time [45].

## Results

**Fig. 1:**
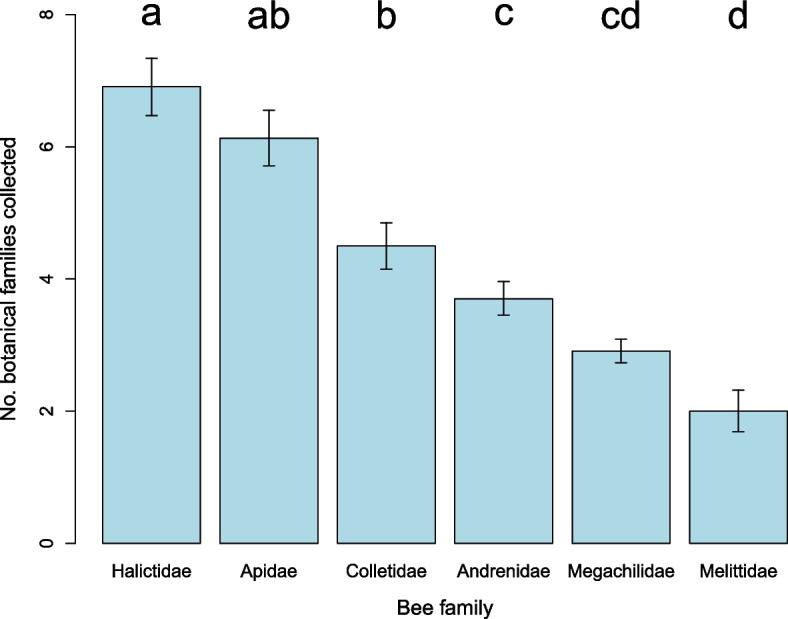
Dietary breadth in the six major bee families (average number of botanical families collected in a standardized sample of 10 pollen loads). Error bars are ±1 standard error of the mean. Different letters above columns indicate significant differences (*p* < 0.05)

In total, published and novel data were available for 860 species of bee, from the six major bee families (Andrenidae *n* = 230, Apidae *n* = 131, Colletidae *n* = 104, Halictidae *n* = 100, Megachilidae *n* = 261, and Melittidae *n* = 34, not including the family Stenotritidae which contains just 21 species) with a total of 24,288 analyzed pollen loads. This represents 4.2% of the approximately 20,759 bee species known globally [46], and 4.8% of the pollen-collecting bee species, excluding the estimated 13% of obligately parasitic bee species [15]. The bee species investigated were found to collect pollen from 119 botanical families (Appendix S2), with the most widely utilized being Asteraceae (22.2% of collected pollen), Fabaceae (15.7%), Brassicaceae (6.9%), Boraginaceae (6.6%), and Rosaceae (6.0%). Across the bee families (Fig. 1), dietary breadth differed meaningfully (Kruskal-Wallis with Dunn’s post-hoc test, χ^2^ = 105.63, *p* < 0.001), with Halictidae (average 6.9 botanical families in a standardized sample of 10 pollen loads) collecting significantly more than the other bee families with the exception of Apidae (average 6.1 botanical families), from which there was no significant difference. Melittidae had the narrowest diets, collecting an average of 2.0 botanical families in a standardized sample of 10 pollen loads, significantly lower than all other bee families with the exception of Megachilidae (average 2.9 botanical families).

**Fig. 2:**
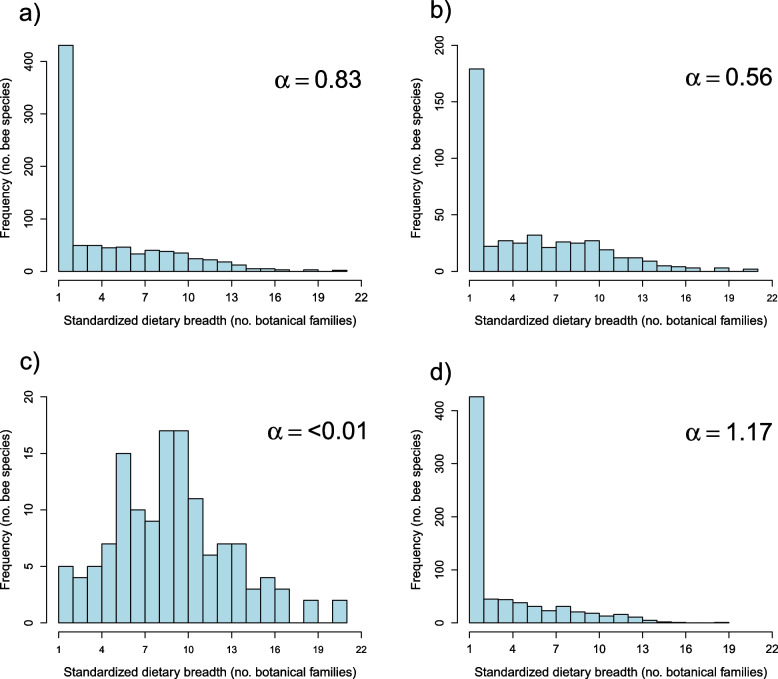
Distribution of diet breadth for **a** all bee species, **b** bee species following taxonomic correction, **c** eusocial bee lineages, and **d** solitary bee lineages; also shown is the shape parameter (α) from the discrete, truncated Pareto distribution

Across all analyzed bee species (*n* = 860), specialization amounted to 431 species (50.1%) collecting between 1 and 2 botanical families in a standardized sample of 10 pollen loads (Fig. 2a). There was a relatively thick and long tail of generalists, with 95 species (11.0%) collecting 10 or more botanical families. This distribution resulted in an *α* value of 0.83, reflecting the relatively low dominance of specialists and a distinct deviation from a truncated Pareto distribution. Exclusion of species with more marginal or irregular supporting data (*n* = 114, see Methods) resulted in a total of 317 species collecting between 1 and 2 botanical families (42.5%), producing an *α* value of 0.75.

**Fig. 3:**
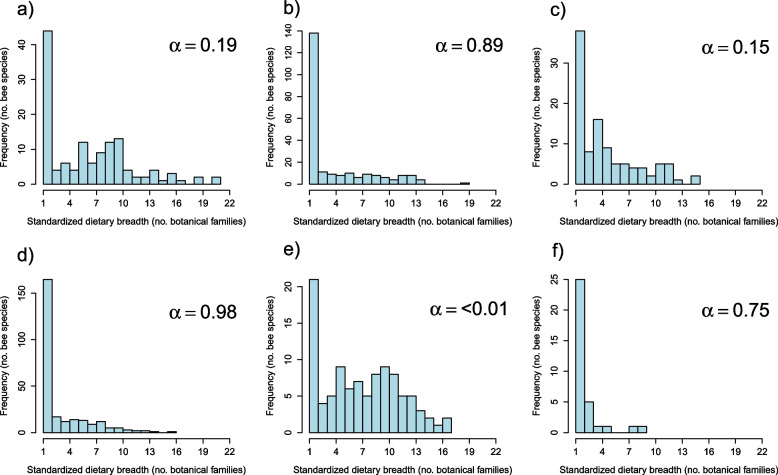
Distribution of diet breadth for **a** family Apidae, **b** family Andrenidae, **c** family Colletidae, and **d** family Megachilidae, **e** family Halictidae, and **f** family Melittidae; also shown is the shape parameter (α) from the discrete, truncated Pareto distribution

For the full dataset, after taxonomic correction (see Methods), the incidence of specialists decreased (*n* = 179, 39.5%), and the incidence of broad generalists increased (*n* = 70, 15.4% collecting 10 or more botanical families), decreasing the *α* value to 0.56 (Fig. 2b). This decreased *α* value was due to the removal of species from of over-represented bee families which happened to display a higher incidence of specialists, specifically Andrenidae (*α* = 0.89; Fig. 3b), Megachilidae (*α* = 0.98; Fig. 3d), and Melittidae (*α* = 0.75; Fig. 3f). In contrast, the unmodified Apidae had a low *α* value of 0.19 (Fig. 3a) and Halictidae had an extremely low *α* value of < 0.01 (Fig. 3e). This extremely low *α* value demonstrates that the distribution of dietary specialization within Halictidae does not conform to a truncated Pareto distribution.

For the species with the broadest diets (Table 1), 14 of the top 20 have been demonstrated to display eusocial behavior in at least part of their range see [47], with the additional species *Lasioglossum mediterraneum* suspected be eusocial due to its close genetic relationship to the eusocial *L. laticeps* [48]. Separation of eusocial lineages (Bombini, Meliponini, Augochlorini, Halictini *s. str.*) produced an extremely low *α* value of < 0.01 for these lineages (Fig. 2c), and a higher *α* value of 1.17 for solitary species (Fig. 2d). For the eusocial lineages, the distribution of diet breadths did not significantly differ from a normal distribution (Shapiro-Wilk test, W = 0.983, *p* = 0.093), and only five species (3.7% of selected eusocial species; all five species were secondarily solitary *Lasioglossum* (*Hemihalictus*) species, see Methods) collected between 1 and 2 botanical families; for solitary lineages 426 species (58.9%) collected between 1 and 2 botanical families.

**Table 1:** The 20 species with the broadest pollen diets after standardization to 10 pollen loads

Family	Species	Social status	No. botanical families collected in 10 pollen loads	Data source
Apidae	*Bombus pratorum*	Eusocial	20.2	44
Apidae	*Melipona mimetica*	Eusocial	20.2	80
Apidae	*Bombus terrestris*	Eusocial	18.9	44
Apidae	*Scaptotrigona* sp.	Eusocial	18.3	80
Andrenidae	*Andrena bicolor*	Solitary	18.1	42
Halictidae	*Lasioglossum morio*	Eusocial	16.8	*Novel data*
Halictidae	*Lasioglossum cupromicans*	Eusocial	16.7	*Novel data*
Apidae	*Bombus impatiens*	Eusocial	16.6	44
Apidae	*Bombus bimaculatus*	Eusocial	15.6	44
Apidae	*Bombus griseocollis*	Eusocial	15.4	44
Apidae	*Bombus pascuorum*	Eusocial	15.3	44
Halictidae	*Lasioglossum laticeps*	Eusocial	15.2	*Novel data*
Megachilidae	*Osmia bicornis*	Solitary	15.0	41
Halictidae	*Lasioglossum mediterraneum*	Probably eusocial	14.9	*Novel data*
Apidae	*Bombus hypnorum*	Eusocial	14.5	44
Colletidae	*Hylaeus hyalinatus*	Solitary	14.3	37
Halictidae	*Lasioglossum calceatum*	Facultatively eusocial	14.3	43
Colletidae	*Hylaeus communis*	Solitary	14.1	37
Apidae	*Bombus perplexus*	Eusocial	13.7	44
Andrenidae	*Andrena rogenhoferi*	Solitary	13.5	89

## Discussion

Though it seems unusual to describe a group of herbivorous insects with 50% of species associated with 1–2 botanical families as containing a relatively ‘low’ percentage of dietary specialists, compared to the Lepidoptera (68–69%) and other herbivorous lineages (76%) [22, 49], bees do appear to have comparatively reduced levels of specialization. This reduced specialization was particularly apparent in the families Apidae and Halictidae that contain social bee lineages [50]. Dietary generalization was so pronounced in the social bee lineages themselves that specialization was almost absent, and the distribution of dietary breadth conformed to a normal distribution, radically departing from the truncated Pareto distribution that characterizes insect herbivory globally. When considering solitary bees alone, their distribution of dietary breadths conforms to the truncated Pareto distribution and they displayed comparable, though typically slightly lower, levels of specialization to other herbivorous insect lineages. As a caveat, it is important to note that it may be inherently easier to detect generalization in bees, since collected pollen can be removed from visible and easy to observe adult females, this pollen representing visits to potentially hundreds of individual flowers. In contrast, in insects whose larvae feed directly on plant material, such larvae must be located and reared (e.g. [22]), potentially overlooking secondary or little-utilized host plants. This potential methodological bias aside, these results pose two main questions; why is dietary generalization so pronounced in lineages containing social species, and why do solitary bees still exhibit a moderate rate of specialization?

When considering the quantitative pollen requirements of developing bee larvae, often requiring the pollen from hundreds of flowers to provision a single larva [51], and also the reduction in fitness associated with travelling long distances to reach specific resources [52], it can be argued that generalization in combination with floral constancy would be an optimal strategy for bees, since the ability to visit a wide variety of flowers in close proximity would provide the greatest quantity for the lowest energy expenditure. The fact that many solitary bees pursue a specialized diet suggests that they are still affected to a certain extent by the same major factors as other herbivorous insects; their observed *α* value of 1.17 is not strongly different from the *α* value of 1.32 observed for non-Lepidopteran herbivores, but considerably lower than the α value of 1.85 observed in Lepidoptera [22].

The principal factor proposed to shape herbivory in insects has been the role of phytochemicals mediating plant-insect co-evolution as secondary defense compounds [17, 19, 53]. Despite the rich literature for other insect groups, the impact of chemical or physical (for example, the structure of the pollen grain exine) protection of pollen to deter excess harvesting and a possible promotion of specialized behavior in bees is much less studied and has mixed support, with initial rejection of this mechanism [54, 55] followed by increased theoretical and empirical support [31, 38, 56, 57]. Additional study shows that levels of alkaloids in Boraginaceae pollen are correlated with concentrations found in the corolla, suggesting spillover rather than active sequestration [58]. In addition, levels of alkaloids were comparatively lower in Boraginaceae species pollinated by pollen-collecting bees, suggesting that mutual relationships with the bees led to a reduction of the alkaloid levels in the pollen. Moreover, although alkaloids had detrimental impacts on larval fitness (though see [59]), botanical lineages with higher alkaloid concentrations in their pollen did not host a greater number of specialized bees [58].

Lastly, because adult bees are mobile, they have the ability to collect pollen from multiple sources before mixing it into the pollen provisions consumed by their larvae. This mixing can dilute and mitigate the negative properties of specific pollen types that cannot be successfully consumed in a pure form [60]. Collectively, these results suggest that whilst there may be clear examples of chemical or physical protection of pollen which does promote or necessitate dietary specialization (e.g. [57]), collectively this may be less important for structuring specialization in bees compared to more directly antagonistic herbivorous insect lineages. The ‘balanced mutual exploitation’ of bees and plants requiring pollination may therefore have contributed to decreased rates of dietary specialization, possibly through reduced levels of secondary compounds in the pollen of bee-pollinated plants.

The likely weaker effect of secondary compounds can be seen in patterns of dietary conservation in bees relative to Lepidoptera. In Lepidoptera, some tribes or even subfamilies feed predominantly on a single or a few closely related botanical families [17, 49], but in bees, such dietary conservation at a tribal level in bees is unknown. Whilst some smaller genera (< 50 species) can display specialization on a single botanical family or genus such as *Systropha* (Halictidae) on Convolvulaceae [61] or *Macropis* (Melittidae) on *Lysimachia* Primulaceae [62], most cases of conserved specialization are transient and concern groups of species or subgenera within genera, even for genera composed predominantly of specialists (e.g. *Chelostoma* (Megachilidae); [39]). Whilst subfamilies, tribes, and genera are artificial taxonomic constructions, and direct one-to-one comparison within this framework is inappropriate, it is clear that the conserved pattern observed in butterflies over long periods of evolutionary time never occurs in bees. For example, members of the subfamily Pierinae across all continents are predominantly associated with the unrelated families Brassicaceae and Capparidaceae as well as a few other families that produce glucosinolates. Ignoring the 36 current genera recognized within Pierinae, this clade is estimated to have arisen 47 million years ago [49], and would thus be comparable in age to subfamilies or tribes within the bees.

If the impact of phytochemical protection of pollen is relatively less important in structuring the dietary choices of bees compared to other insect herbivores, what other factors can explain the lower but still meaningful level of specialization in bees, particularly solitary species? For mobile, central-place foragers such as bees, a variety of factors driving or maintaining specialization have been proposed [24, 63], specifically those of spatially or temporally dense resources, low travel costs, high inherent differences in resource quality (thus including phytochemical mediated resource quality), and high difficulties associated with resource procurement and utilization (e.g. the difficulty associated with extracting pollen from flowers with complex morphologies [64]), all favoring a specialized diet [43].

A central concept uniting these factors is that of foraging efficiency. For solitary bees, whose adult stages are active for a short period of time usually measured in days or weeks rather than months, they must spend hours visiting flowers in order to provision a single brood cell, whereas an insect herbivore with free-living larvae can lay dozens of eggs in a fraction of this time. The time taken to gather resources is therefore of critical importance for foraging bees. Importantly, bees are unusual as herbivores in that they can only collect plant pollen when it is available during flowering, a necessarily much shorter period of time than is available for herbivores that consume vegetative plant parts. For bees, resource density is thus temporal as well as spatial, and factors that shape flowering period determine resource availability in time. In this context, high spatial resource density [63] favors specialization (the ‘predictable plethora’ hypothesis [54, 65, 66]; see also [67]), and a long flight period relative to a specific resource favors generalization [24]. Empirical data shows that solitary *Andrena* species that are active for only a short period have broader diets in less seasonal environments, but that species with equivalent flight periods have diets that are more constrained and more specialized in more seasonal environments [43, 68]. In contrast, the diets of *Lasioglossum* species with long flight periods are essentially unaffected by seasonality, as these flight periods are sufficiently long to have access to all flowering resources throughout their activity period [43]. Indeed, this pattern can be seen even within *Andrena*, as bivoltine *Andrena* species that *de facto* have longer flight periods than univoltine *Andrena* species display the widest diets within this genus [42, 68]. This long activity period not only explains why social bee lineages can have such generalized diets, but why they must be generalized; a necessary step in the evolution of eusociality is the production of overlapping generations [2], and overlapping generations mean extended activity periods during which any individual resource will be relatively sparsely distributed and hence unavailable.

In addition to resource density, resource quality remains important, though this also includes floral structure in addition to phytochemical composition of pollen. Although many plants have an open structure, allowing easy access to pollen and nectar, others have complex flower morphologies (e.g. bilateral symmetry, poricidal anthers, nototriby) that render pollen more challenging to access. For complex flowers, specialists can display greater foraging efficiency due to lower handling time per flower compared to generalists [69]. This adaptation can also be morphological, for example through the gain of specialized pollen-collecting hairs to groom pollen from nototribic anthers [64], or behavioral through the use of buzz pollination [70]. Within solitary bees, multiple radiations can be seen within clades that utilize morphologically complex bilaterally-symmetrical flowers, implying an inherent difficulty in processing these flower shapes compared to radially symmetrical flowers and evolutionary advantages once this difficulty is overcome (e.g. [71–73]). Whilst this may constrain pollen collection in solitary lineages in which females forage as independent individuals, within eusocial bumble bees, behavioral specialization can occur at the individual worker level for specific time periods [74, 75], maximizing resource utilization at the level of the colony and allowing use of a wide variety of floral morphologies. Social bees are therefore able to escape some of the foraging constraints experienced by solitary bees due to their longer activity periods and behavioral adaptations, such as learning to manipulate complex flowers [76], even if this is less efficient when compared to specialists.

Finally, it must first be discussed to what extent these data presented here are representative of bee diversity globally, both in terms of selected species and biogeographical regions. As in any such analysis, certain lineages will be over-sampled, and others undersampled. For example, detailed pollen data meeting our selection criteria were available for only 14 *Megachile* species, despite this being the third most diverse bee genus globally with c. 1400 species distributed throughout almost all global habitat types [71]. In part, this lack of data comes from a paucity of studies that have quantified pollen use by bees in tropical areas at the species level using pollen load data, and thus our dataset contains primarily Holarctic species. Since insect herbivore specialization (as well as species diversity) is known to increase at lower latitudes [22, 77], could our dataset be underestimating specialization in bees? This is considered unlikely, because bees are unusual in that they display a bimodal pattern of diversity, with the greatest richness at mid-latitudes, with comparatively mediocre diversity in the tropics [78]. In combination, bee dietary specialization is likely to be lowest in tropical areas and highest in seasonally dry regions (summarized in [24]; note, this is based primarily on observational rather than palynological data). For example, tropical bee faunas contain typical bee genera from tribes such as Centridini and Euglossini that are known or expected to be predominantly widely polylectic (e.g. [79, 80]). Tropical bee faunas also contain a large stingless bee element (Meliponini), a group of obligately eusocial bees that are expected to be overwhelmingly generalized (e.g. [81–83]) but which were under-sampled in our dataset. Only data for two of the c. 540 species of Meliponini [46] were suitable for inclusion in our dataset due a methodological preference for researchers working on Meliponini to analyze stored pollen removed from nests rather than pollen removed from individual foragers, but both of these species were in the top four most generalized species studied here. Additional sampling in tropical areas is therefore considered unlikely to increase estimates of dietary specialization in bees, and the lack of a latitudinal diversity gradient in itself is likely to be a contributing factor to the diverging pattern of specialization seen between bees and other herbivorous insects.

## Conclusions

In this empirical context, it is clear that bees remain subject to some constraints that shape their collection of pollen and their resulting dietary breadths; a slight majority of the species studied here are specialized on a single botanical family. However, these constraints appear to be comparatively weaker than those seen in other lineages of herbivorous insects, and bees collectively appear to have elevated rates of generalization, strongly driven by the high levels of generalization observed in social bee lineages. We posit that these elevated rates are driven by two evolutionary processes: firstly, the at least partly mutual relationship between bees and flowers may have led to a reduced role of phytochemicals in structuring bee diet breadth compared to other herbivorous lineages. Secondly, behavioral adaptations such as elongated activity periods combined with behavioral adaptations allow social bees to collect pollen from a wide variety of plants. Though the most important factors structuring dietary specialization in bees appear to differ from many other herbivorous insects, with a comparatively reduced importance of plant chemistry and comparatively increased importance of phenology and foraging efficiency, solitary bees show a surprisingly similar overall pattern of dietary breadth to these other lineages.

## Methods

In contrast to host-plant records generated for other herbivorous insect lineages that come predominantly from rearing observations and experiments, understanding host plant use by bees comes from the quantification of pollen loads carried by adults. As bee larvae only have access to the pollen provisions laid up for them by their mother (or siblings or nestmates in the case of eusocial bees), pollen carried by adult bees is therefore representative of the larval diet. The greater the number of analyzed individuals, the higher the degree of confidence that the full dietary breadth has been captured, as bees can visit multiple flower species during the course of a single foraging bout or between bouts.

Quantitative data documenting bee pollen preferences was assembled based on a thorough review of available literature and the generation of novel data. Publications were selected based on the following criteria: 1) bees are determined to the species level (only six species were determined to morphospecies in our dataset); 2) pollen data consists of the analysis of specimen-by-specimen pollen loads OR analysis of nest provisions; 3) pollen taxa are identified to at least the botanical family level; 4) results are presented as an overall percentage per botanical family. Publications were considered from anywhere in the world, with no geographic framework. For pollen load analysis, a minimum threshold of 10 analyzed pollen loads was set, with the following caveats. Analysis of nest provisions (e.g. [84]) was considered to be the equivalent of 10 analyzed pollen loads for species demonstrated to be specialized, but was not used for species found to be generalized due to the impossibility of relating nest contents to a specific number of pollen loads. A total of 13 specialized bee species in our dataset were classified based solely on nest content analysis. For some publications, the number of pollen loads analyzed was not specified (e.g. [85]), but where the species was conclusively found to be a specialist the data were included with an assumed number of 10 analyzed pollen loads. For palynological analyses that did not meet the 10 load threshold but were supported by field observations (in the same or from other literature sources) consistent with oligolecty (specialization on a single botanical family), these species were included and assumed to have 10 analyzed pollen loads. Due to this potential bias towards specialists, additional analyses were run with these species excluded.

Following these criteria, a total of 96 primary literature sources were identified that present quantitative palynological data on bee pollen use (Appendix S1). In addition, novel and previously unpublished data on wild bee pollen preferences were generated following established methodology (e.g. [36, 38, 41–44]). Briefly, pinned specimens of identified bee species were relaxed. The size of pollen loads on individual bees was estimated, ranging from a full load to a one-eighth load. Pollen was removed from scopal hairs using an entomological pin and transferred to a drop of water on a microscope slide. Slides were heated to remove excess water, fuchsin-stained glycerin jelly was added and the slide was sealed with a coverslip. The percentage of the load composed of different plant species was estimated along three randomly selected lines across the cover slip at a magnification of × 400. The percentage of the load was estimated by the relative area of the slide occupied by each plant species, rather than the absolute number of grains see [38, 86]. Pollen species representing < 2% of the load were excluded from further analysis because their presence might have arisen from contamination. The percentages of pollen collected were corrected according to the overall size of each load to give a final weighting. Pollen loads were identified to the lowest taxonomic level possible using a reference collection assembled from local floras. In the context of this project, only the botanical-family level data were used.

Due to differing sample sizes between bee species, a rarefaction procedure was used to standardize sample sizes to 10 pollen loads, chosen at random without replacement 1000 times package *vegan* [87]. As this procedure is designed for use on integer data, and pollen data is measured as a percentage, the pollen load data were first transformed. For example, with a sample size of 14, the percentage of pollen collected from each botanical family was multiplied by the sample size to give a whole pollen load equivalent, e.g. 40% becomes 5.6 pollen loads, 10% becomes 1.4 pollen loads. These values were all multiplied by 10 and rounded to the nearest whole number to give an integer equivalent that was used in the rarefaction procedure. In order to facilitate comparison with other herbivorous lineages, the frequency distribution of these standardized diets was used to calculate a shape parameter (*α*; 22). The methodology and R code of [22] was used to calculate this metric.

As the dataset contains some species supported by a mixture of sources (a limited number of analyzed pollen loads combined with literature data) or unclear data (the number of analyzed pollen loads is not stated), a smaller data subset was created, excluding specialized species whose dietary breadth was supported by either i) an analysis of nest contents, ii) an unspecified number of analyzed pollen loads, or iii) whose sample size was below the 10 pollen load threshold. This amounted to 114 species. The remaining species (*n* = 746) were analyzed to ensure that use of these data on specialized bees did not have an impact on the observed results from the analysis of all bee species.

Due to an uneven distribution of empirical pollen studies globally, there are few studies from tropical regions in general, and relatively few studies from the bee families Apidae (2.2% of global species represented in our dataset) and Halictidae (2.2% of global species) specifically, bee families which also dominate tropical faunas. In contrast, Melittidae (16.6% of global species), Andrenidae (7.6% of global species), Megachilidae (6.3% of global species), and Colletidae (3.8% of global species) were over-represented in our dataset relative to Apidae and Halictidae. A simple taxonomic correction was made, decreasing the number of species in these four families in order to achieve a uniform 2.2% representation. To do this, species within each of these families were sorted alphabetically and removed randomly (final sample *n* = 453, Andrenidae *n* = 66, Apidae *n* = 131, Colletidae *n* = 60, Halictidae *n* = 100, Megachilidae *n* = 91, and Melittidae *n* = 5).

In order to test for the effects of sociality, eusocial and solitary lineages were separated. Eusocial lineages included two tribes within the Apidae, Bombini (*Bombus*) and Meliponini (represented here by *Melipona* and *Scaptotrigona*). It is important to note that we did not include any *Apis* species (honey bees) which are known to have extremely broad diets, but which are usually studied by analyzing combined pollen samples from pollen traps, thus these samples representing hundreds of individual pollen loads (e.g. [88]). Such data are thus incomparable with the individual load-by-load data used here to standardize dietary breadth measurements, though *Apis* are clearly dietary hyper-generalists. Within the Halictidae, sociality in the subfamily Halictinae is more complex due to the high diversity of social behaviors and transitions between social and solitary behavior [89]. Due to the lack of robust data for every species in this dataset, a conservative position was taken, and all members of the tribes Augochlorini and Halictini *s. str.* (e.g. not including the species around *Agapostemon*) were included as social species. All analyses were conducted in R version 3.6.3.

### Supplementary Information

**Additional file 1.**

**Additional file 2.**

## Data Availability

Data presented in this manuscript are a mixture of those already published and publicly available, with those publications properly cited in this submission, and novel unpublished data that are available from Figshare https://figshare.com/s/76b80dd1fbe365e9fc5b (Appendix S1. Supporting primary and secondary literature; Appendix S2. Summary of palynological data by botanical family).
