# Supplementary material for: Elevated rates of dietary generalization in eusocial lineages of the secondarily herbivorous bees
Source: BMC Ecol Evol. 2023 Nov 20;23:67. doi: 10.1186/s12862-023-02175-1 (PMC10662511; doi:10.1186/s12862-023-02175-1)
Supplement: Supplementary file 1 — Additional file 1. [file 12862_2023_2175_MOESM1_ESM.docx]

**Appendix I. Supporting primary and secondary literature for Wood et al. “Escaping the constraints of herbivory: elevated rates of dietary generalization in eusocial lineages of the secondarily herbivorous bees”.**

**Primary literature sources**

[1] Bennet, B., and M.D. Breed. 1985. The nesting biology, mating behavior, and foraging ecology of *Perdita opuntiae* (Hymenoptera: Andrenidae). Journal of the Kansas Entomological Society 58:185-194.

[2] Carvalho, A.T., and C. Schlindwein. 2011. Obligate association of an oligolectic bee and a seasonal aquatic herb in semi-arid north-eastern Brazil. Biological Journal of the Linnean Society 102:355-368.

[3] Cripps, C., and R.W. Rust. 1989. Pollen foraging in a community of *Osmia* bees (Hymenoptera: Megachilidae). Environmental Entomology 18:582-589.

[4] Dorchin, A., A. Shafir, F.H. Neumann, D. Langgut, N.J. Vereecken, and I. Mayrose. Bee flowers drive macroevolutionary diversification in long-horned bees. Proceedings of the Royal Society B 288:20210533.

[5] Dos Santos, I.A., and D. Wittmann. 2000. Legitimate pollination of the tristylous flowers of *Eichhornia azurea* (Pontederiaceae) by *Ancyloscelis* *gigas* bees (Anthophoridae, Apoidea). Plant Systematics and Evolution 223:127-137.

[6] Estes, J.R., and R.W. Thorp. 1975. Pollination ecology of *Pyrrhopappus carolinianus* (Compositae). American Journal of Botany 62:148-159.

[7] Ferton, C. (1892) Sur les moeurs de quelques Hyménoptères de la Provence du genre *Osmia*. Actes de la Société Linnéenne de Bordeaux 45:231-240.

[8] Gaona, F.P., A. Guerrero, E. Gusmán, and C.I. Espinosa. 2019. Pollen resources used by two species of stingless bees (Meliponini) in a tropical dry forest of southern Ecuador. Journal of Insect Science 19: 1-5.

[9] Glaum, P. and Wood, T.J., J.R. Morris, and F.S. Valdovinos. 2021. Phenology and flowering overlap drive specialisation in plant-pollinator networks. Ecology Letters 24:2648-2659.

[10] González-Varo, J.P., F.J. Ortiz-Sánchez, and M. Vila. 2016. Total bee dependence on one flower species despite available congeners of similar floral shape. PLoS One 11:e0163122.

[11] Gotlieb, A., G. Pisanty, J.G. Rozen, A. Müller, G. Röder, C. Sedivy, and C.J. Praz. 2014. Nests, floral preferences, and immatures of the bee *Haetosmia vechti* (Hymenoptera: Megachilidae: Osmiini). American Museum Novitates 3808:1–20.

[12] Haider, M., S. Dorn, C. Sedivy, and A. Müller. 2014. Phylogeny and floral hosts of a predominantly pollen generalist group of mason bees (Megachilidae: Osmiini). Biological Journal of the Linnean Society 111:78-91.

[13] Herrmann, M., F. Burger, A. Müller, and S. Tischendorf. 2003. Verbreitung, Lebensraum und Biologie der Furchen biene *Lasioglossum pallens* (Brülle 1832) und ihrer Kuckucksbiene *Sphecodes majalis* Perez 1903 in Deutschland (Hymenoptera, Apidae, Halictinae). Carolinea 61:133-144.

[14] Houston, T., B.B. Lamont, S. Radford, and S.G. Errington. 1993. Apparent Mutualism between *Verticordia nitens* and *V. aurea* (Myrtaceae) and their Oil-ingesting Bee Pollinators (Hymenoptera: Colletidae). Australian Journal of Botany 41:369-380.

[15] Hurd, P.D., and E.G. Linsley. 1964. The Squash and Gourd Bees—Genera *Peponapis* Robertson and *Xenoglossa* Smith—Inhabiting America North of Mexico (Hymenoptera: Apoidea). Hilgardia, 35:375-477.

[16] Immelman, K., and C. Eardley. 2000. Gathering of grass pollen by solitary bees (Halictidae, *Lipotriches*) in South Africa. Mitteilungen aus dem Museum für Naturkunde in Berlin. Zoologische Reihe 76:263-268.

[17] Kuhlmann, M., and C. Eardley. 2011. Pollen resources of non-*Apis* bees in southern Africa. In: Evolution of Plant–Pollinator Relationships, S. Patiny ed. Cambridge University Press, pp. 439-456.

[18] Kuhlmann, M., and K. Timmermann. 2009. Nest Architecture and floral Hosts of the South African Monolectic solitary Bee *Othinosmia* (*Megaloheriades*) *schultzei* (Hymenoptera: Megachilidae). Entomologia Generalis 32:1-9.

[19] Kuhlmann, M., and K. Timmermann. 2011. Nest architecture of the monolectic South African solitary bee, *Samba* (*Prosamba*) *spinosa* Eardley (Hymenoptera: Apoidea: Melittidae). African Entomology 19:141-145.

[20] Larkin, L.L., J.L. Neff, and B.B. Simpson. 2008. The evolution of a pollen diet: Host choice and diet breadth of *Andrena* bees (Hymenoptera: Andrenidae). Apidologie 39:133-145.

[21] Manderey, K., J. Kosuch, and J. Schuberth. 2008. Untersuchungsergebnisse zum Artstatus von *Andrena decipiens* Schenck, 1861, *Andrena flavilabris* Schenck, 1874, und ihrem gemeinsamen Brutparasiten *Nomada stigma* Fabricius, 1804 (Hymenoptera: Apidae). Nachrichtenblatt der Bayerischen Entomologen 57:30-41.

[22] Maubecin, C.C., L. Boero, and A.N. Sérsic. 2020. Specialisation in pollen collection, pollination interactions and phenotypic variation of the oil-collecting bee *Chalepogenus cocuccii*. Apidologie 51:710-723.

[23] Michez, D., and S. Patiny. 2005. World revision of the oil-collecting bee genus *Macropis* Panzer 1809 (Hymenoptera: Apoidea: Melittidae) with a description of a new species from Laos. Annales de la Société Entomologique de France 41:15-28.

[24] Michez, D., S. Patiny, P. Rasmont, K. Timmermann, and N.J. Vereecken. 2008. Phylogeny and host-plant evolution in Melittidae s.l. (Hymenoptera: Apoidea). Apidologie 39 146-162.

[25] Michez, D., C. Eardley, M. Kuhlmann, K. Timmermann, and S. Patiny. 2010. The bee genera *Haplomelitta* and *Samba* (Hymenoptera : Anthophila : Melittidae): phylogeny, biogeography and host plants. Invertebrate Systematics 24:327-347.

[26] Milet-Pinheiro, P., and Schlindwein, C. 2010. Mutual reproductive dependence of distylic *Cordia leucocephala* (Cordiaceae) and oligolectic *Ceblurgus longipalpis* (Halictidae, Rophitinae) in the Caatinga. Annals of Botany 106:17-27.

[27] Miranda, E.A., I.N. Lima, C.A. Oi, M.M. López-Uribe, M.A. Del Lama, B.M. Freitas, and C.I. Silva. 2021. Overlap of ecological niche breadth of *Euglossa cordata* and *Eulaema nigrita* (Hymenoptera, Apidae, Euglossini) accessed by pollen loads and species distribution modeling. Neotropical Entomology 50:197-207.

[28] Moisan-Deserres, J., M. Girard, M. Chagnon, and V. Fournier. 2014. Pollen Loads and Specificity of Native Pollinators of Lowbush Blueberry. Horticultural Entomology 107:1156-1162.

[29] Müller, A., and P. Westrich. 2023. Morphological specialisation for primary nectar robbing in a pollen specialist mining bee (Hymenoptera, Andrenidae). Journal of Hymenoptera Research 95:215-230.

[30] Müller, A., and N. Bansac. 2004. A specialized pollen-harvesting device in western palaearctic bees of the genus *Megachile* (Hymenoptera, Apoidea, Megachilidae). Apidologie 35:329-337.

[31] Müller, A., and T.L. Griswold. 2017. Osmiine bees of the genus *Haetosmia* (Megachilidae, Osmiini): biology, taxonomy and key to species. Zootaxa 4358:351- 364.

[32] Müller, A., and M. Kuhlmann. 2008. Pollen hosts of western palaearctic bees of the genus *Colletes* (Hymenoptera: Colletidae): the Asteraceae paradox. Biological Journal of the Linnean Society 95:719-733.

[33] Müller, A., and V. Mauss. 2016. Palaearctic *Hoplitis* bees of the subgenera *Formicapis* and *Tkalcua* (Megachilidae, Osmiini): biology, taxonomy and key to species. Zootaxa 4127:105-120.

[34] Müller, A., and H. Richter. 2018. Dual function of *Potentilla* (*Rosaceae*) in the life history of the rare boreoalpine osmiine bee *Hoplitis* (*Formicapis*) *robusta* (Hymenoptera, Megachilidae). Alpine Entomology 2:139-147.

[35] Müller, A., and V. Trunz. 2018. Palaearctic osmiine bees of the genera *Hofferia* and *Stenoheriades* (Megachilidae, Osmiini): biology, taxonomy and key to species. Zootaxa 3765:175-186.

[36] Müller, A. 1994. Die Bionomie der in leeren Schneckengehäusen nistenden Biene *Osmia spinulosa* (Kirby 1802) (Hymenoptera, Megachilidae). Veröffentlichungen für Naturschutz und Landschaftspflege Baden-Württemberg 68/69:291-334.

[37] Müller, A. 1995. Morphological specializations in central european bees for the uptake of pollen from flowers with anthers hidden in narrow corolla tubes (Hymenoptera: Apoidea). Entomologia Generalis 20:43-57.

[38] Müller, A. 1996a. Host-plant specialization in Western Palearctic anthidine bees (Hymenoptera: Apoidea: Megachilidae). Ecological Monographs 66:235-257.

[39] Müller, A. 1996b. Convergent evolution of morphological specializations in Central European bee and honey wasp species as an adaptation to the uptake of pollen from nototribic flowers (Hymenoptera, Apoidea and Masaridae). Biological Journal of the Linnean Society 57: 235-252.

[40] Müller, A. 2014a. Palaearctic *Hoplitis* bees of the subgenera *Chlidoplitis* and *Megahoplitis* (Megachilidae, Osmiini): biology, taxonomy and key to species. Zootaxa 3765:161-174.

[41] Müller, A. 2014b. Palaearctic *Hoplitis* bees of the subgenus *Stenosmia* (Megachilidae, Osmiini): biology, taxonomy and key to species. Zootaxa 3765:301-316.

[42] Müller, A. 2015. Nest architecture and pollen hosts of the boreoalpine osmiine bee species *Hoplitis* (*Alcidamea*) *tuberculata* (Hymenoptera, Megachilidae). Journal of Hymenoptera Research 47:53-64.

[43] Müller, A. 2018a. Pollen host selection by predominantly alpine bee species of the genera *Andrena*, *Panurginus*, *Dufourea*, *Megachile*, *Hoplitis* and *Osmia* (Hymenoptera, Apoidea). Alpine Entomology 2:101-113.

[44] Müller, A. 2018b. Palaearctic Osmia bees of the subgenus *Hoplosmia* (Megachilidae, Osmiini): biology, taxonomy and key to species. Zootaxa 4415:297-329.

[45] Müller, A. 2020. Palaearctic Osmia bees of the subgenera *Hemiosmia*, *Tergosmia* and *Erythrosmia* (Megachilidae, Osmiini): biology, taxonomy and key to species. Zootaxa 4778:201-236.

[46] Müller, A. 2022. New Moroccan bee species of the tribe Osmiini (Hymenoptera: Apoidea: Megachilidae). Zootaxa 5188:233-263.

[47] Müller, A. 2023. The hidden diet – examination of crop content reveals distinct patterns of pollen host use by Central European bees of the genus *Hylaeus* (Hymenoptera, Colletidae). Alpine Entomology 7:21-35.

[48] Müller, A., C.J. Praz, and A. Dorchin. 2018. Biology of Palaearctic *Wainia* bees of the subgenus *Caposmia* including a short review on snail shell nesting in osmiine bees (Hymenoptera, Megachilidae). Journal of Hymenoptera Research 65:61-89.

[49] Müller, A., R. Prosi, C.J. Praz, and H. Richter. 2019. Nesting in bark – the peculiar life history of the rare boreoalpine osmiine bee *Osmia* (*Melanosmia*) *nigriventris* (Hymenoptera, Megachilidae). Alpine Entomology 3:105-119.

[50] Neff, J.L., and B.N. Danforth. 1991. The Nesting and Foraging Behavior of *Perdita texana* (Cresson) (Hymenoptera: Andrenidae). Journal of the Kansas Entomological Society 64:394-405.

[51] Neff, J.L., and L.L. Larkin. 2002. *Andrena chaparralensis* New Species, a New Vernal Bee Associated with Asteraceae on the South Texas Plains (Hymenoptera: Apoidea: Andrenidae). Journal of the Kansas Entomological Society 75:268-273.

[52] Neff, J.L., and J.G. Rozen. 1995. Foraging and Nesting Biology of the Bee *Anthemurgus passiflorae* (Hymenoptera: Apoidea), Descriptions of Its Immature Stages, and Observations on Its Horal Host (Passifloraceae). American Museum Novitates 3138:1-19.

[53] Neff, J.L., and B.B. Simpson. 1997. Nesting and Foraging Behavior of *Andrena* (*Callandrena*) *rudbeckiae* Robertson (Hymenoptera: Apoidea: Andrenidae) in Texas. Journal of the Kansas Entomological Society 70:100-113.

[54] Nilsson, L.A. 2009. The type material of Swedish bees (Hymenoptera, Apoidea) III. Entomologisk Tidskrift 130:43-59.

[55] Norden, B.B., K.V. Krombein, M.A. Deyrup, and J.P. Edirisinghe. 2003. Biology and behavior of a seasonally aquatic bee, *Perdita* (*Alloperdita*) *floridensis* Timberlake (Hymenoptera: Andrenidae: Panurginae). Journal of the Kansas Entomological Society 76:236-249.

[56] O’Niell, R.P., and K.M. O’Niel. 2011. Pollen load composition and size in the leafcutting bee *Megachile rotundata* (Hymenoptera: Megachilidae). Apidologie 42:223-233.

[57] Parker, F.D. 1978. Biology of the Bee Genus *Proteriades* Titus (Hymenoptera: Megachilidae). Journal of the Kansas Entomological Society 51:145-173.

[58] Parker, F.D., and V.J. Tependino. 1982. A Nest and Pollen-Collection Records of *Osmia sculleni* Sandhouse, a Bee with Hooked Hairs on the Mouthparts (Hymenoptera: Megachilidae). Journal of the Kansas Entomological Society 55:329-334.

[59] Pauly, A., K. Timmermann, and M. Kuhlmann. 2008. Description of a new interesting species from South Africa, *Evylaeus* (*Sellalictus*) *fynbosensis* n.sp. (Hymenoptera Apoidea Halictidae). Journal of Afrotropical Zoology 4:85-91.

[60] Pick, R.A., and C. Schlindwein. 2011. Pollen partitioning of three species of Convolvulaceae among oligolectic bees in the Caatinga of Brazil. Plant Systematics and Evolution 293:147-159.

[61] Popov, V.B. 1952a. On related species of *Tridentosmia*. Zoologicheskiy Zhurnal (Moscow) 31:183-190.

[62] Popov, V.B. 1952b. Bee fauna (Hymenoptera, Apoidea) of south-western Turkmenia and its landscape distribution. Trudy Zoologicheskogo Instituta Akademii Nauk SSSR (Leningrad [St. Petersburg]) 10:61-117.

[63] Popov, V.B. 1967. The bees (Hymenoptera, Apoidea) of Middle Asia and their associations with angiosperm plants. Trudy Zoologiceskzo Instituta Akademija Nauk SSSR (Leningrad [St. Petersburg]) 38: 11-329.

[64] Praz, C.J., D. Genoud, K. Vaucher, D. Bénon, J. Monks, and T.J. Wood. 2022. Unexpected levels of cryptic diversity in European bees of the genus *Andrena* subgenus *Taeniandrena* (Hymenoptera, Andrenidae): implications for conservation. Journal of Hymenoptera Research 91:375-428.

[65] Praz, C.J., A. Müller, and D. Genoud. 2019. Hidden diversity in European bees: *Andrena amieti* sp. n., a new Alpine bee species related to *Andrena bicolor* (Fabricius, 1775) (Hymenoptera, Apoidea, Andrenidae). Alpine Entomology 3:11-38.

[66] Prosi, R., H. Wiesbauer, and A. Müller. 2016. Distribution, biology and habitat of the rare European osmiine bee species *Osmia* (*Melanosmia*) *pilicornis* (Hymenoptera, Megachilidae, Osmiini). Journal of Hymenoptera Research 52:1-36.

[67] Quiroz-Garcia, D.L., E. Martinez-Hernandez, R. Palacios-Chavez, and N.E. Galindo- Miranda. 2001. Nest Provisions and Pollen Foraging in Three Species of Solitary Bees (Hymenoptera: Apidae) from Jalisco, Mexico. Journal of the Kansas Entomological Society 74:61-69.

[68] Romankova, T.G. 1985. A new species of the bee genus *Osmia* (Hymenoptera, Megachilidae) from the Far East. Zoologicheskiy Zhurnal 64: 942-944.

[69] J.G. Rozen. 1971. Biology and Immature Stages of Moroccan Panurgine Bees (Hymenoptera, Apoidea). American Museum Novitates 2457:1-37.

[70] R.W. Rust. 1980. The Biology of Ptilothrix bombiformis (Hymenoptera: Anthophoridae). Journal of the Kansas Entomological Society 53:427-436.

[71] R.W. Rust, G. Cambon, J.T. Grossa, and B.E. Vassière. 2004. Nesting Biology and Foraging Ecology of the Wood-Boring Bee *Lithurgus chrysurus* (Hymenoptera: Megachilidae). Journal of the Kansas Entomological Society 77:269-279.

[72] Schlindwein, C., and C.F. Martins. 2000. Competition between the oligolectic bee *Ptilothrix plumata* (Anthophoridae) and the flower closing beetle *Pristimerus calcaratus* (Curculionidae) for floral resources of *Pavonia cancellata* (Malvaceae). Plant Systematics and Evolution 224:183-194.

[73] Schlindwein, C., and P.C.R. Medeiros. 2005. Pollination in *Turnera subulata* (Turneraceae): Unilateral reproductive dependence of the narrowly oligolectic bee *Protomeliturga turnerae* (Hymenoptera, Andrenidae). Flora 201:178-188.

[74] Schlindwein, C., and D. Wittmann. 1995. Specialized solitary bees as effective pollinators of south Brazilian species of *Notocactus* and *Gymnocalycium*. Bradleya 13:25-34.

[75] Schlindwein, C., and D. Wittmann 1997. Micro foraging routes of *Bicolletes pampeana* (Colletidae) and bee induced pollen presentation in *Cajophora arechavaletae* (Loasaceae). Botanica Acta 110: 177-183.

[76] Sedivy, C., C.J. Praz, A. Müller, A. Widmer, and S. Dorn. 2008. Patterns of host-plant choice in bees of the genus *Chelostoma*: the constraint hypothesis of host-range evolution in bees. Evolution 62:2487-2507.

[77] Sedivy, C., S. Dorn, A. Widmer, and A. Müller. 2013. Host range evolution in a selected group of osmiine bees (Hymenoptera: Megachilidae): the Boraginaceae-Fabaceae paradox. Biological Journal of the Linnean Society 108:35-54.

[78] Sipes, S.D. 2001. Phylogenetic relationships, taxonomy, and evolution of host choice in *Diadasia* (Hymenoptera: Apoidea). PhD Dissertation. Logan, UT: Utah State University.

[79] Sipes, S.D., and V.J. Tepedino. 2005. Pollen-host specificity and evolutionary patterns of host switching in a clade of specialist bees (Apoidea: *Diadasia*). Biological Journal of the Linnean Society 86:487-505.

[80] Siriani-Oliveira, S., R. Oliveira, and C. Schlindwein. 2018. Pollination of *Blumenbachia amana* (Loasaceae): flower morphology and partitioned pollen presentation guarantee a private reward to a specialist pollinator. Biological Journal of the Linnean Society 124:479-491.

[81] Siriani-Oliveira, S., I. Cerceau, and C. Schlindwein. 2019. Specialised protagonists in a plant–pollinator interaction: the pollination of *Blumenbachia insignis* (Loasaceae). Plant Biology 22:167-176.

[82] Tepedino, V.J. 2003. What's in a Name? The Confusing Case of the Death Camas Bee, *Andrena astragali* Viereck and Cockerell (Hymenoptera: Andrenidae). Journal of the Kansas Entomological Society 76:194-197.

[83] Tepedino, V.J., B.A. Bradley, and T.L. Griswold. 2008. Might Flowers of Invasive Plants Increase Native Bee Carrying Capacity? Intimations From Capitol Reef National Park, Utah. Natural Areas Journal 28:44-50.

[84] Tepedino, V.J., T.L. Griswold, J.E. Freilich, and P. Shephard. 2011. Specialist and generalist bee visitors of an endemic beardtongue (*Penstemon caryi*: Plantaginaceae) of the Big Horn mountains, Wyoming. Western North American Naturalist 71:523- 528.

[85] Timmermann, K., and M. Kuhlmann. 2008. The biology of a *Patellapis (s. str.)* species (Hymenoptera: Apoidea: Halictidae): sociality described for the first time in this bee genus. Apidologie 39:189-197.

[86] Westrich, P. 1989. Die wildbienen Baden-Württembergs. Eugen Ulmer, Stuttgart.

[87] Westrich, P. 2008. *Andrena sardoa* Lepeletier, 1841, eine streng oligolektische, auf Asphodelus (Asphodelaceae) spezialisierte Bienenart (Hymenoptera, Apidae) der westlichen Mediterraneis. Entomologische Nachrichten und Berichte 52:133-137.

[88] Westrich, P. 2010. Untersuchungen zum Blütenbesuch von Bienen (Hymenoptera, Apidae) an *Ornithogalum* s. l. (Milchstern, Hyacinthaceae). Eucera 3:1-17.

[89] Westrich, P. 2014. Beitrag zur Diskussion über den taxonomischen Status von *Andrena rosae* Panzer 1801 (Hymenoptera, Apidae). Eucera 8:1-11.

[90] Westrich, P., F. Gusenleitner, and F. Amiet. 1997. *Andrena afrensis* Warncke 1967, eine für Mitteleuropa neue Bienen-Art (Hymenoptera, Apidae). Linzer biologische Beiträge 29:1167-1174.

[91] Wood, T.J., and I. Cross. 2017. *Camptopoeum* (*Camptopoeum*) *baldocki* spec. nov., a new panurgine bee species from Portugal and a description of the male of *Flavipanurgus* *fuzetus* Patiny (Andrenidae: Panurginae). Zootaxa 4254:285-293.

[92] Wood, T.J., and R. Le Divelec. 2022. Cryptic Diversity Revealed in A Revision of West Palaearctic *Nomiapis* and *Systropha* (Hymenoptera: Halictidae). Diversity 14:920.

[93] Wood, T.J., and S.P.M. Roberts. 2017. An assessment of historical and contemporary diet breadth in polylectic *Andrena* bee species. Biological Conservation 215: 72-80.

[94] Wood, T.J., and S.P.M. Roberts. 2018. Constrained patterns of pollen use in Nearctic [92 *Andrena* (Hymenoptera: Andrenidae) compared to their Palearctic counterparts. Biological Journal of the Linnean Society 124:732-746.

[95] Wood, T.J. 2023. The genus *Andrena* Fabricius, 1775 in the Iberian Peninsula (Hymenoptera, Andrenidae). Journal of Hymenoptera Research 96:241-484.

[96] Wood, T.J., J. Gibbs, K.K. Graham, and R. Isaacs. 2019. Narrow pollen diets are associated with declining Midwestern bumble bee species. Ecology 100:e02697.

[97] Wood, T.J. 2022. Two new overlooked bee species from Spain (Hymenoptera: Anthophila: Andrenidae, Apidae). Journal of Hymenopterology 10:1-12.

[98] Wood, T.J., G. Ghisbain, P. Rasmont, D. Kleijn, I. Raemakers, C.J. Praz, M. Killewald, J. Gibbs, K. Bobiwash, M. Boustani, B. Martinet, and D. Michez. 2021. Global patterns in bumble bee pollen collection show phylogenetic conservation of diet. Journal of Animal Ecology 90:2421-2430.

[99] Wood, T.J., G. Ghisbain, D. Michez, and C.J. Praz. 2021b. Revisions to the faunas of *Andrena* of the Iberian Peninsula and Morocco with the descriptions of four new species (Hymenoptera: Andrenidae). European Journal of Taxonomy 758:147-193.

[100] Wood, T.J., S. Patiny, and S. Bossert. 2022. An unexpected new genus of panurgine bees (Hymenoptera, Andrenidae) from Europe discovered after phylogenomic analysis. Journal of Hymenoptera Research 89:183-210.

**Secondary literature sources**

[101] Amiet, F., M. Herrmann, A. Müller, and R. Neumeyer. 2004. Fauna Helvatica 9. Apidae 4. *Anthidium*, *Chelostoma, Coelioxys, Dioxys, Heriades, Lithurgus, Megachile, Osmia, Stelis*. Centre Suisse de Cartographie de la Faune (CSCF)/Schweizerische Entomologische Gesellschaft (SEG), Neuchâtel, 274 pp.

[102] Gibbs, J., J.S. Ascher, M.G. Rightmyer, and R. Isaacs. 2017. The bees of Michigan (Hymenoptera: Apoidea: Anthophila), with notes on distribution, taxonomy, pollination, and natural history. Zootaxa 4352:1-160.

[103] Hurd, P.D. 1979. Superfamily Apoidea. In: Krombein, K.V., P.D. Hurd Jr., D.R. Smith, and B.D. Burks (Eds.), Catalog of Hymenoptera in America North of Mexico. Smithsonian Institution Press, Washington, D.C., pp. 1741-2209.

[104] LaBerge, W.E. and D.W. Ribble. 1972. A revision of the bees of the genus *Andrena* of the Western Hemisphere. Part IV. *Gonandrena*, *Geissandrena*, *Parandrena*, *Pelicandrena*. Transactions of the American Entomological Society 98:271-358.

[105] LaBerge, W.E. 1967. A revision of the bees of the genus *Andrena* of the Western Hemisphere. Part I. *Callandrena* (Hymenoptera: Andrenidae). Bulletin of the University of Nebraska State Museum 7:1-316.
